# Correction: Taokaew, S.; Kriangkrai, W. Chitinase-Assisted Bioconversion of Chitinous Waste for Development of Value-Added Chito-Oligosaccharides Products. *Biology* 2023, *12*, 87

**DOI:** 10.3390/biology12050689

**Published:** 2023-05-08

**Authors:** Siriporn Taokaew, Worawut Kriangkrai

**Affiliations:** 1 Department of Materials Science and Bioengineering, School of Engineering, Nagaoka University of Technology, Nagaoka, Niigata 940-2188, Japan; 2 Department of Pharmaceutical Technology, Faculty of Pharmaceutical Sciences, Naresuan University, Phitsanulok 65000, Thailand

In the original publication [1], there was a mistake in the published reference [3], which was as follows:

3. Vickers, N.J. Animal Communication: When I’m Calling You, Will You Answer Too? *Curr. Biol.* **2017**, *27*, R713–R715. 

The corrected reference [3] is as below: 

3. Venugopal, V. Valorization of Seafood Processing Discards: Bioconversion and Bio-Refinery Approaches. *Front. Sustain. Food Syst.* **2021**, *5*, 611835. https://doi.org/10.3389/fsufs.2021.611835.

The authors state that the scientific conclusions are unaffected. This correction was approved by the Academic Editor. The original publication has also been updated.
